# A Review of Recent Research Progress on the Astragalus Genus

**DOI:** 10.3390/molecules191118850

**Published:** 2014-11-17

**Authors:** Xiaoxia Li, Lu Qu, Yongzhe Dong, Lifeng Han, Erwei Liu, Shiming Fang, Yi Zhang, Tao Wang

**Affiliations:** 1 Tianjin State Key Laboratory of Modern Chinese Medicine, 312 Anshanxi Road, Nankai District, Tianjin, 300193, China; E-Mails: huifeidedouzi@yeah.net (X.L.); qululuhan88@163.com (L.Q.); dongyongzhe44@hotmail.com (Y.D.); 2 Tianjin Key Laboratory of TCM Chemistry and Analysis, Institute of Traditional Chinese Medicine, Tianjin University of Traditional Chinese Medicine, 312 Anshan Road, Nankai District, Tianjin 300193, China; E-Mails: hanlifeng_1@163.com (L.H.); liuwei628@hotmail.com (E.L.); fang_shiming@163.com (S.F.)

**Keywords:** traditional Chinese medicines, Astragalus genus, phytochemistry, biological activities, analyses

## Abstract

Astragalus L., is one of the largest genuses of flowering plants in the Leguminosae family. Roots of *A. membranaceus* Bge. *var*. *mongholicus* (Bge.) Hsiao, *A. membranaceus* (Fisch.) Bge. and its processed products are listed in the China Pharmacopeia for “qi deficiency” syndrome treatment. However, more and more researches on other species of Astragalus have been conducted recently. We summarize the recent researches of Astragalus species in phytochemistry and pharmacology. More than 200 constituents, including saponins and flavonoids, obtained from 46 species of Astragalus genus were collected for this article. In pharmacological studies, crude extracts of Astragalus, as well as isolated constituents showed anti-inflammatory, immunostimulant, antioxidative, anti-cancer, antidiabetic, cardioprotective, hepatoprotective, and antiviral activities. The goal of this article is to provide an overview of chemical and pharmacological studies on the Astragalus species over the last 10 years, which could be of value to new drug or food supplement research and development.

## 1. Introdution

Astragalus L., is one of the largest genuses of flowering plants in the Leguminosae family. As annual or perennial herbs, subshrubs, or shrubs, the plants of Astragalus L. are widely distributed throughout the temperate and arid regions. So far, the genus has been estimated to contain 2000–3000 species and more than 250 taxonomic sections in the world [1,2,3].

Some species of Astragalus in Asia are a source of the economically important natural product, gum tragacanth. In addition, the dried roots of some species grown in East Asia are well used in Traditional Chinese Medicines (TCM) as antiperspirants, diuretics, and tonics for a wide array of diseases such as empyrosis, nephritis, diabetes mellitus, hypertension, cirrhosis, leukaemia, and uterine cancer [4,5]. For example, the root of *A. membranceus* (Fisch.) Bge. var. *mongholicus* (Bge.) Hsiao (Radix Astragali) is a precious medicine in TCM, which has the properties of intensifying phagocytosis of reticuloendothelial systems, stimulating pituitary-adrenal cortical activity, and restoring depleted red blood cell formation in bone marrow. Also, it is famed for its antimicrobial, antiperspirant, anti-inflammatory, diuretic and tonic effects [6]. Some plants in the Astragalus genus are well known for their pharmacological properties, particularly hepatoprotective, immunostimulant, and antiviral activities [7]. While, the most common use of this genus is as forage for livestock and wild animals, some plants in this genus have been recognized as being used in foods, medicines, cosmetics, as substitutes for tea or coffee, or as sources of vegetable gums.

Saponins, flavonoids, and polysaccharides are believed to be the principle active constituents of Astragalus [8]. It also includes components such as anthraquinones, alkaloids, amino acids, *β*-sitosterol, and metallic elements.

Here, we have undertaken this review in an effort to summarize the available literatures on these promising bioactive natural products. The review focuses on the phytochemistry, biological activities, and analysis of the Astragalus genus.

## 2. Phytochemistry

**Figure 1:**
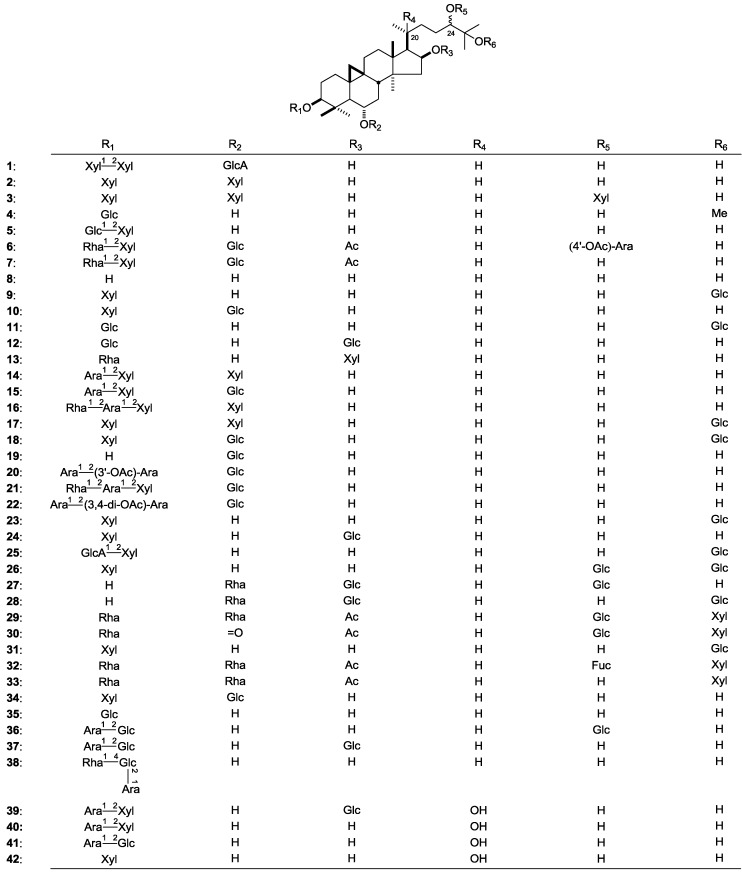

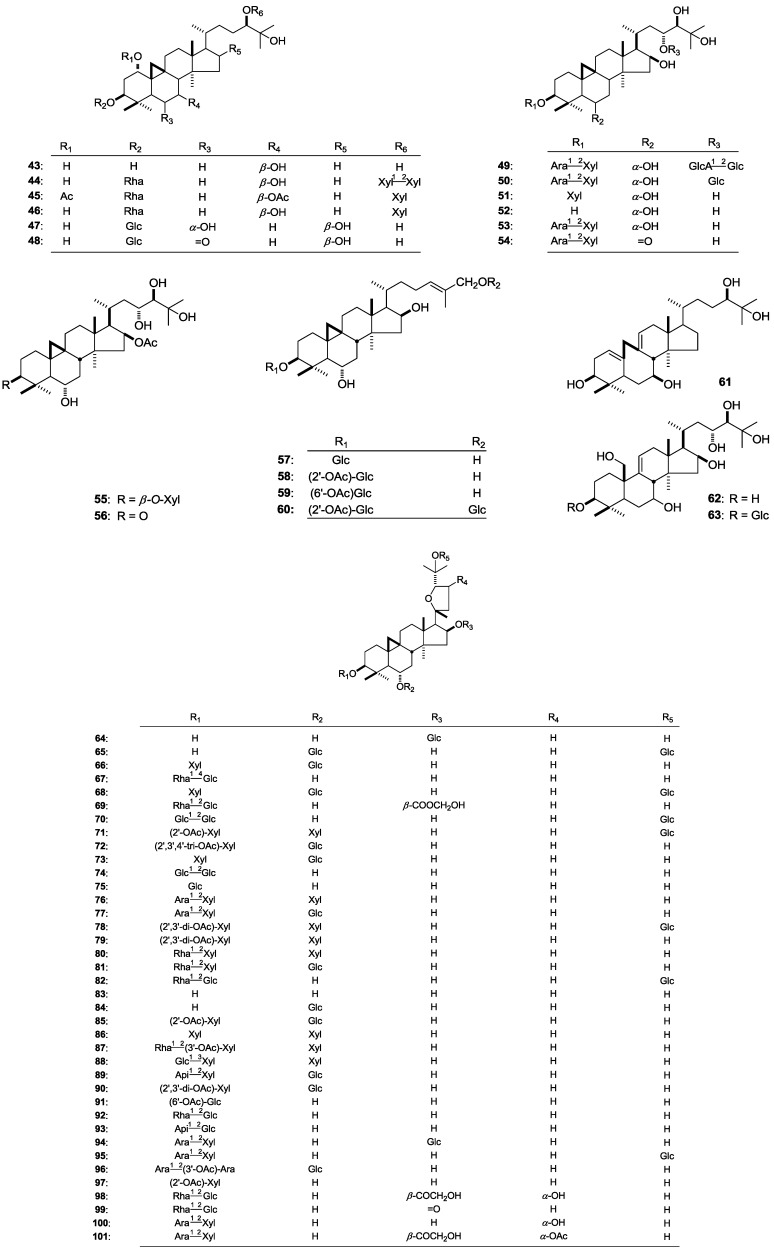

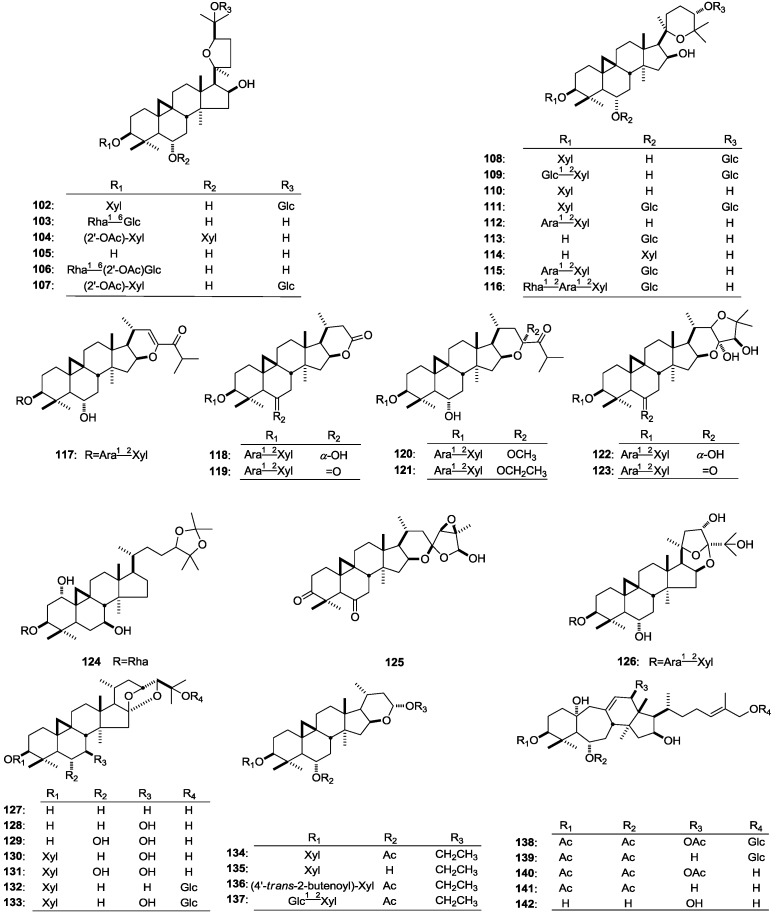
The structures of compounds **1**–**142** obtained from the Astragalus genus.

**Figure 2:**
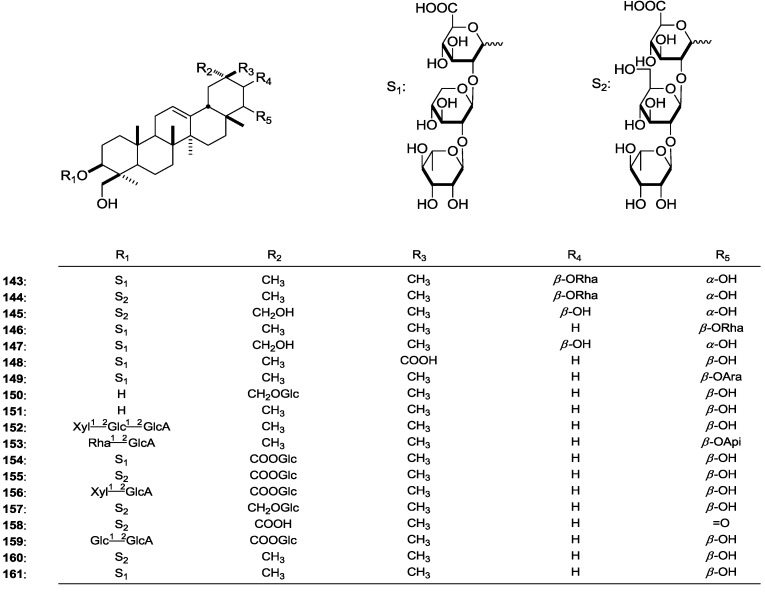
The structures of compounds **143**–**161** obtained from the Astragalus genus.

**Figure 3:**
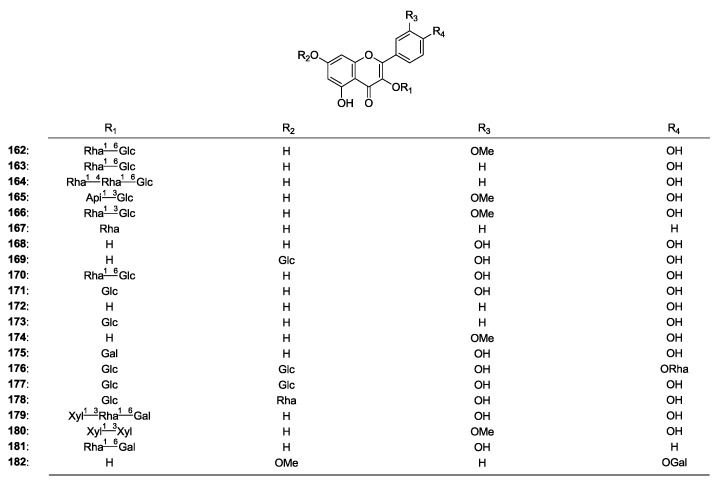

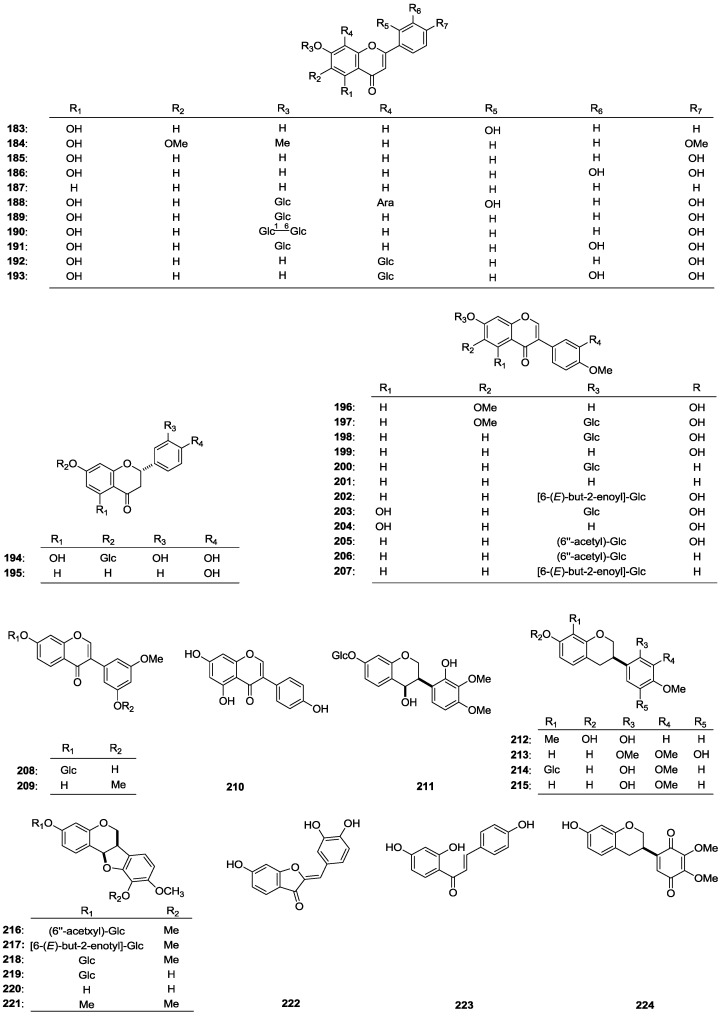
The structures of compounds **162**–**224** obtained from the Astragalus genus.

**Figure 4:**
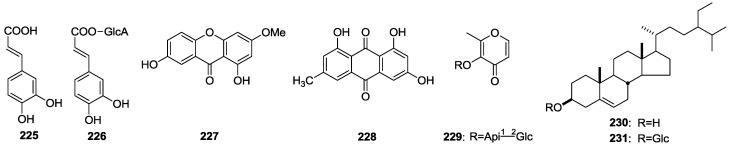
The structures of compounds **225**–**231** obtained from the Astragalus genus.

As the summarized results shown in Table 1, Table 2 and Table 3 and Figure 1, Figure 2, Figure 3 and Figure 4, 46 kinds of Astragalus species have been studied for their chemical constituents in recent years [9,10,11,12,13,14,15,16,17,18,19,20,21,22,23,24,25,26,27,28,29,30,31,32,33,34,35,36,37,38,39,40,41,42,43,44,45,46,47,48,49,50,51,52,53,54,55,56,57,58,59,60,61,62,63,64,65,66,67,68,69,70,71,72,73,74,75,76,77,78,79]. Also there have been more than 200 constituents obtained from them. Though the studies were for different species, the chemical compositions in Astragalus genus appeared highly uniform, which mainly include terpenoids, flavonoids, and polysaccharides. The interesting compounds, such as terpenoids and flavonoids are always in free or glycosidic forms. Meanwhile, we found that about 40 percent of the composition researches were focused on the aerial parts of Astragalus.

### 2.1. Saponins

Saponin is the major chemical constituent type in the Astragalus genus. Cycloartane- and oleanane-type saponins from it showed interesting biological properties.

#### 2.1.1. Cycloartane-Type Saponins

The plants of Astragalus genus have been proved to be the richest source of cycloartane-type saponins, possessing cardiotonic, hypocholesteremic, anti-depressive and antiblastic actions as well as immunomodulatory activity [7]. This promising spectrum of pharmacological effects led researchers to further search for structurally interesting cycloartane glycosides from the genus. Until now, more than 140 kinds of cycloartane-type saponins have been identified (Table 1, Figure 1). The main substituted sugar groups in them are *β*-d-glucopyranosyl (Glc), *β*-d-xylopyranosyl (Xyl), *α*-l-rhamnopyranosyl (Rha), or *α*-l-arabinopyranosyl (Ara). Additionally, *β*-d-glucuronopyranosyl (GlcA), *β*-d-fucopyranosyl (Fuc), *β*-d-apiofuranosyl (Api) and acetyl (Ac) groups were also found in cycloartane glycosides obtained from the Astragalus genus.

**Table 1:** Cycloartane-type triterpenoids from the Astragalus genus (**1**–**142**).

	Compound’s Name	Species Resource	Parts Used	Reference
1	3*-O*-[*β*-d-Xylopyranosyl(1→2)-*β*-d-xylopyranosyl]-6-*O*-*β*-d-glucuronopyranosyl-3*β*,6*α*,16*β*,24(*S*),25-pentahydroxyxyxloartane	*A.* *erinaceus*	whole plant	[9]
2	Hareftoside A	*A.* *erinaceus*	whole plant	[9]
		*A*. *hareftae*	whole plant	[10]
3	Hareftoside B	*A*. *hareftae*	whole plant	[10]
4	Cycloquivinoside A	*A.* *chivensis*	aerial parts	[11]
5	Astramembranosides B	*A. membranaceus*	roots	[12]
6	3-*O*-[*α*-l-Rhamnopyranosyl(1→2)-*β*-d-xylopyranosyl]-6-*O*-*β*-d-glucopyranosyl-24-*O*-*α*-(4'-*O*-acetoxy)-l-arabinopyranosyl-16-*O*-acetoxy-3*β*,6*α*,16*β*,24*S*,25-pentahydroxycycloartane	*A. wiedemannianus*	whole plant	[13]
7	3-*O*-[*α*-l-Rhamnopyranosyl(1→2)-*β*-d-xylopyranosyl]-6-*O*-*β*-d-glucopyranosyl-24-*O*-*α*-l-arabinopyranosyl-16-*O*-acetoxy-3*β*,6*α*,16*β*,24(*S*),25-pentahydroxycycloartane	*A. wiedemannianus*	whole plant	[13]
8	Cyclocanthogenin	*A. unifoliolatus*	epigeal parts	[14]
		*A. chivensis*	aerial parts	[15]
9	3-*O*-*β*-d-Xylopyraosyl-24(*S)*-cycloart-3*β*,6*α*,16*β*,24,25-pentaol-25-*O*-*β*-d-glucopyranoside	*A. ernestii*	roots	[16]
		*A. amblolepis*	roots	[17]
10	Cyclocanthoside E	*A*. *hareftae*	whole plant	[10]
		*A. oleifolius*	lower stem parts	[18]
		*A. caucasicus*	leave *s*	[19]
11	Cyclochivinoside B	*A. chivensis*	aerial parts	[15]
12	Cyclochivinoside C	*A. chivensis*	aerial parts	[20]
13	Caspicuside I	*A. caspicus*	roots	[6]
14	Oleifoliosides A	*A. oleifolius*	lower stem parts	[18]
15	Oleifoliosides B	*A. oleifolius*	lower stem parts	[18]
16	3-*O*-[*α-*l-Rhamnopyranosyl(1→2)-*α*-l-arabinopyranosyl(1→2)-*β*-d-xylopyranosyl]-6-*O*-*β*-d-xylopyranosyl-3*β*,6*α*,16*β*,24(*S*),25-pentahydroxycycloartane	*A. aureus*	whole plant	[21]
17	3,6-di-*O*-*β*-d-Xylopyranosyl-25-*O*-*β*-d-glucopyranosyl-3*β*,6*α*,16*β*,24(*S*),25-pentahydr-oxycycloartane	*A. aureus*	whole plant	[21]
18	3-*O*-*β*-d-Xylopyranosyl-6,25-di-*O*-*β*-d-glucopyranosyl-3*β*,6*α*,16*β*,24(*S*),25-pentahydroxycycloartane	*A. aureus*	whole plant	[21]
19	6-*O*-*β*-d-Glucopyranosyl-3*β*,6*α*,16*β*,24(*S*),25-pentahydroxycycloartane	*A. aureus*	whole plant	[21]
20	3-*O*-[*α*-l-Arabinopyranosyl(1→2)-*O*-3-acetoxy-*α*-l-arabinopyranosyl]-6-*O*-*β*-d-glucopyranosyl-3*β*,6*α*,16*β*,24(*S*),25-pentahydroxycycloartane	*A. icmadophilus*	whole plant	[22]
21	3-*O*-[*α*-l-Rhamnopyranosyl(1→2)-*O*-*α*-l-arabinopyranosyl(1→2)-*O*-*β*-d-xylopyranosyl]-6-*O*-*β*-d-glucopyranosyl-3*β*,6*α*,16*β*,24(*S*),25-pentahydroxycycloartane	*A. icmadophilus*	whole plant	[22]
22	3-*O*-[*α*-l-Arabinopyranosyl(1→2)-*O*-3,4-diacetoxy-*α*-l-arabinopyranosyl]-6-*O*-*β*-d-glucopyranosyl-3*β*,6*α*,16*β*,24(*S*),25-pentahydroxycycloartane	*A. icmadophilus*	whole plant	[22]
23	3-*O*-*β*-d-Xylopyranosyl-25-*O*-*β*-d-glucopyranosyl-3*β*,6*α*,16*β*,24(*S*),25-pentahydroxycycloartane	*A. ernestii*	roots	[16]
		*A. amblolepis*	roots	[17]
24	3-*O*-*β*-d-Xylopyranosyl-16-*O*-*β*-d-glucopyranosyl-3*β*,6*α*,16*β*,24(*S*),25-pentahydroxycycloartane	*A. amblolepis*	roots	[17]
25	3-*O*-[*β*-d-Glucuronopyranosyl(1→2)-*β*-d-xylopyranosyl]-25-*O*-*β*-d-glucopyranosyl-3*β*,6*α*,16*β*,24(*S*),25-pentahydroxy-cycloartane	*A. amblolepis*	roots	[17]
26	3-*O*-*β*-d-Xylopyranosyl-24,25-di-*O*-*β*-d-glucopyranosyl-3*β*,6*α*,16*β*,24(*S*),25-pentahydr-oxy-cycloartane	*A. amblolepis*	roots	[17]
27	6-*O*-*α*-l-Rhamnopyranosyl-16,24-di-*O*-*β*-d-glucopyranosyl-3*β*,6*α*,16*β*,24*(S*),25-pentahydroxy cycloartane	*A. amblolepis*	roots	[17]
28	6-*O*-*α*-l-Rhamnopyranosyl-16,25-di-*O*-*β*-d-glucopyranosyl-3*β*,6*α*,16*β*,24(*S*),25-pentahydroxy cycloartane	*A. amblolepis*	roots	[17]
29	Cicerosides A	*A. cicer*	aerial parts	[7]
30	Cicerosides B	*A. cicer*	aerial parts	[7]
31	Cycloascidoside	*A. ernestii*	roots	[16]
		*A. amblolepis*	roots	[17]
		*A. mucidus*	aerial parts	[23]
32	Eremophiloside A	*A. eremophilus*	aerial parts	[24]
33	Eremophiloside B	*A. eremophilus*	aerial parts	[24]
34	Cycloascidoside A	*A. mucidus*	aerial parts	[25]
35	Cyclounifoliside C	*A. unifoliolatus*	epigeal parts	[14]
		*A. chivensis*	aerial parts	[15]
36	3-*O*-[*α*-l-Arabinopyranosyl(1→2)-*β*-d-glucopyranosyl]-24-*O*-*β*-d-glucopyranosyl-3*β*,6*α*,16*β*,24(*R*),25-pentahydroxycycloartane	*A. stereocalyx*	roots	[26]
37	3-*O*-[*α*-l-Arabinopyranosyl(1→2)-*β*-d-glucopyranosyl]-16-*O*-*β*-d-glucopyranosyl-3*β*,6*α*,16*β*,24(*R*),25-pentahydroxycycloartane	*A. stereocalyx*	roots	[26]
38	3-*O*-{*α*-l-Rhamnopyranosyl(1→4)-[*α*-l-arabinopyranosyl(1→2)]-*β*-d-glucopyranosyl}-3*β*,6*α*,16*β*,24(*R*),25-pentahydroxycycloartane	*A. stereocalyx*	roots	[26]
39	3-*O*-[*α*-l-Arabinopyranosyl(1→2)-*β*-d-xylopyranosyl]-16-*O*-*β*-d-glucopyranosyl-3*β*,6*α*,16*β*,20(*S*),24(*R*),25-hexahydroxycycloartane	*A. stereocalyx*	roots	[26]
		*A. halicacabus*	whole plant	[27]
		*A. campylosema* Boiss. *subsp. campylosema*	roots	[28]
40	3-*O*-[*α*-l-Arabinopyranosyl(1→2)-*β*-d-xylopyranosyl]-3*β*,6*α*,16*β*,20(*S*),24(*R*),25-hexahydroxycycloartane	*A. stereocalyx*	roots	[26]
41	3-*O*-[*α*-l-Arabinopyranosyl(1→2)-*β*-d-glucopyranosyl]-3*β*,6*α*,16*β*,20(*S*),24(*R*),25-hexahdroxycycloartane	*A. stereocalyx*	roots	[26]
42	3-*O*-*β*-d-Xylopyranosyl-3*β*,6*α*,16*β*,20(*S*),24(*R*),25-hexahydroxycycloartane	*A. schottianus*	roots	[29]
43	Cyclomacrogenin B	*A. macropus*	roots	[30,31]
44	Cyclomacroside E	*A. macropus*	roots	[32]
45	Cyclomacroside B	*A. macropus*	roots	[33]
46	Cyclomacroside D	*A. macropus*	roots	[31]
47	Mongholicoside A	*A. membranace* (Fisch.) Bge. *var. mongholicus* (Bge.)	aerial parts	[34]
48	Mongholicoside B	*A. membranace* (Fisch.) Bge. *var. mongholicus* (Bge.)	aerial parts	[34]
49	Askendoside K	*A. taschkendicus*	roots	[35]
50	Askendoside H	*A. taschkendicus*	roots	[36]
51	Cycloorbicoside D	*A. orbiculatus*	aerial parts	[37,38]
52	Cycloorbigenin C	*A. taschkendicus*	roots	[35,36]
		*A. orbiculatus*	aerial parts	[37,38]
53	Eremophiloside C	*A. eremophilus*	aerial parts	[24]
54	Eremophiloside D	*A. eremophilus*	aerial parts	[24]
55	Bicusposide F	*A. bicuspis*	whole plant	[39]
56	Bicusposide E	*A. bicuspis*	whole plant	[39]
57	Kahiricoside II	*A. kahiricus*	aerial parts	[40]
58	Kahiricoside III	*A. kahiricus*	aerial parts	[40]
59	Kahiricoside IV	*A. kahiricus*	aerial parts	[40]
60	Kahiricoside V	*A. kahiricus*	aerial parts	[40]
61	Secomacrogenin B	*A. macropus*	roots	[41]
62	Orbigenin	*A. orbiculatus*	aerial parts	[37,38]
63	Orbicoside	*A. orbiculatus*	aerial parts	[37,38]
64	16-*O*-*β*-d-Glucopyranosyl-3*β*,6*α*,16*β*,25-tetrahydroxy-20(*R*),24(*S*)-epoxycycloartane	*A*. *hareftae*	whole plant	[10]
65	Astramembranosides A	*A. membranaceus*	roots	[12]
66	Cyclosiversioside F	*A. oldenburgii*	aerial parts	[42]
67	Astraverrucin IV	*A. oldenburgii*	aerial parts	[42]
68	Astragaloside VII	*A. oldenburgii*	aerial parts	[42]
		*A. dissectus*	roots and stems	[43]
		*A. membranace* (Fisch.) Bge. *var. mongholicus* (Bge.) hisao	roots	[44]
69	3-*O*-[*α*-l-Rhamnopyranosyl(1→2)-*β*-d-glucopyranosyl]-16-*O*-hydroxyacetoxy-3*β*,6*α*,16*β*,25-tetrahydroxy-20(*R*),24(*S*)-epoxycycloartane	*A. angustifolius*	whole plant	[45]
70	CyclolehmanosideC	*A. lehmannianus*	aerial parts	[46]
71	Armatoside II	*A. armatus*	roots	[47]
72	Acetylastragaloside I	*A. baibutensis*	roots	[48]
73	Astragaloside III	*A. illyricus*	roots	[49]
		*A. membracaceus*	roots	[50]
74	Cyclounifolioside B	*A. illyricus*	roots	[49]
75	Astraverrucin I	*A. illyricus*	roots	[49]
76	Trigonoside II	*A. armatus*	roots	[47]
		*A. halicacabus*	whole plant	[27]
77	Trojanoside H	*A. stereocalyx*	roots	[26]
		*A. armatus*	roots	[47]
78	Armatoside I	*A. armatus*	roots	[47]
79	Cyclosieversioside A	*A. sieversianus*	roots	[51]
80	Cyclosieversioside G	*A. sieversianus*	roots	[51]
81	Cyclosieversioside H	*A. sieversianus*	roots	[51]
82	3-*O*-[*α*-l-Rhamnopyranosyl(1→2)-*β*-d-glucopyranosyl]-25-*O*-*β*-d-glucopyranosyl-20(*R*),24(*S*)-epoxy-3*β*,6*α*,16*β*,25-tetrahydroxycycloartane	*A. wiedemannianus*	whole plant	[13]
83	Cyclosiversigenin	*A. orbiculatus*	aerial parts	[52]
84	Brachyoside B	*A. wiedemannianus*	whole plant	[13]
85	Astragaloside II	*A*. *hareftae* *A. wiedemannianus*	whole plant whole plant	[10] [13]
86	Astrasieversianin X	*A. wiedemannianus*	whole plant	[13]
87	Astrasieversianin IX	*A. sieversianus*	roots	[51]
88	Caspicuside II	*A. caspicus*	roots	[6]
89	Baibutoside	*A. baibutensis*	roots	[48]
90	Astragalosides I	*A. baibutensis*	roots	[48]
		*A. sieversianus*	roots	[51]
91	Astraverrucin VII	*A. verrucosus*	aerial parts	[53]
92	Cycloaraloside D (Peregrinoside II)	*A. verrucosus*	aerial parts	[53]
		*A. angustifolius*	whole plant	[45]
93	Cycloaraloside C (Astrailienin A)	*A. verrucosus*	aerial parts	[53]
94	(20*R*,24*S*)-3-*O*-[*α*-l-Arabinopyranosyl(1→2)-*β*-d-xylopyranosyl]-20,24-epoxy-16-*O*-*β*-d-glucopyranosyl-3*β*,6*α*,16*β*,25-tetrahydroxycycloartane	*A. halicacabus*	whole plant	[27]			
95	3-*O*-[*α*-l-Arabinopyranosyl(1→2)-*β*-d-xylopyranosyl]-25-*O*-*β*-d-glucopyranosyl-3*β*,6*α*,16*β*,25-tetrahydroxy-20(*R*),24(*S*)-epoxycycloartane	*A. campylosema* Boiss*. subsp. campylosema*	roots	[28]			
96	3-*O*-[*α*-l-Arabinopyranosyl(1→2)-*O*-3-acetoxy-*α*-l-arabinopyranosyl]-6-*O*-*β*-d-glucopyranosyl-3*β*,6*α*,16*β*,25-tetrahydroxy-20(*R*),24(*S*)-epoxycycloartane	*A. icmadophilus*	whole plant	[22]			
97	20(*R*),24(*S*)-Epoxycycloartane-3*β*,6*α*,16*β*,25-tetraol-3-*β*-*O*-d-(2-*O*-acetyl)-xylopyranoside	*A. bicuspis*	whole plant	[39]			
98	3-*O*-[*α*-l-Rhamnopyranosyl(1→2)-*β*-d-glucopyranosyl]-16-*O*-hydroxyacetoxy-3*β*,6*α*,16*β*,23*α*,25-pentahydroxy-20(*R*),24(*S*)-epoxycycloartane	*A. angustifolius*	whole plant	[45]			
99	3-*O*-[*α*-l-Rhamnopyranosyl(1→2)-*β*-d-glucopyranosyl]-3*β*,6*α*,25-trihydroxy-20(*R*),24(*S*)-epoxycycloartane-16-one	*A. angustifolius*	whole plant	[45]			
100	3-*O*-[*α*-l-Arabinopyranosyl(1→2)-*β*-d-xylopyranosyl]-3*β*,6*α*,16*β*,23*α*,25-pentahydroxy-20(*R*),24(*S*)-epoxycycloartane	*A. campylosema* Boiss. *subsp. campylosema*	roots	[28]			
101	3-*O*-[*α*-l-Arabinopyranosyl(1→2)-*β*-d-xylopyranosyl]-16-*O*-hydroxyacetoxy-23-*O*-acetoxy-3*β*,6*α*,16*β*,23*α*,25-pentahydroxy-20(*R*),24(*S*)-epoxycycloartane	*A. campylosema* Boiss. *subsp. campylosema*	roots	[28]			
102	Cyclogaleginoside E	*A. galegiformis*	stems	[54]			
103	Cycloascualoside D	*A. galegiformis*	stems	[55]			
104	Cyclogaleginoside C	*A. galegiformis*	stems	[55]			
105	Cyclogalegigenin	*A. galegiformis*	stems	[54,55,57]			
		*A. caucasicus*	leaves	[56]			
106	Cycloascauloside A	*A. caucasicus*	leaves	[56]			
107	Cyclogaleginoside D	*A. galegiformis*	stems	[57]			
108	20(*R*),25-Epoxy-3-*O*-*β*-d-xylopyranosyl-24-*O*-*β*-d-glucopyranosyl-3*β*,6*α*,16*β*,24*α*-tetrahydroxycycloartane	*A. schottianus*	roots	[29]
109	20(*R*),25-Epoxy-3-*O*-[-*β*-d-glucopyranosyl(1→2)]-*β*-d-xylopyranosyl-24-*O*-*β*-d-glucopyranosyl-3-*β*,6*α*,16*β*,24*α*-tetrahydroxycycloartane	*A. schottianus*	roots	[29]
110	Hareftoside C	*A*. *hareftae*	whole plant	[10]
111	Cylotrisectoside	*A. dissectus*	roots and stems	[43]
112	3-*O*-[*α*-l-Arabinopyranosyl(1→2)-*β*-d-xylopyranosyl]-3*β*,6*α*,16*β*,24*α*-tetrahydroxy-20(*R*),25-epoxycycloartane	*A. aureus*	whole plant	[21]
113	6-*O*-*β*-d-Glucopyranosyl-3*β*,6*α*,16*β*,24*α*-tetrahydroxy-20(*R*),25-epoxycycloartane	*A. aureus*	whole plant	[21]
114	6-*O*-*β*-d-Xylopyranosyl-3*β*,6*α*,16*β*,24*α*-tetrahydroxy-20(*R*),25-epoxycycloartane	*A. aureus*	whole plant	[21]
115	3-*O*-[*α*-l-Arabinopyranosyl(1→2)-*O*-*β*-d-xylopyranosyl]-6-*O*-*β*-d-glucopyranosyl-3*β*,6*α*,16*β*,24*α*-tetrahydroxy-20(*R*),25-epoxycycloartane	*A. icmadophilus*	whole plant	[22]
116	3-*O*-[*α*-l-Rhamnopyranosyl(1→2)-*O*-*α*-l-arabinopyranosyl(1→2)-*O*-*β*-d-xylopyranosyl]-6-*O*-*β*-d-glucopyranosyl-3*β*,6*α*,16*β*,24*α*-tetrahydroxy-20(*R*),25-epoxycycloartane	*A. icmadophilus*	whole plant	[22]
117	Eremophiloside G	*A. eremophilus*	aerial parts	[24]
118	Eremophiloside E	*A. eremophilus*	aerial parts	[24]
119	Eremophiloside F	*A. eremophilus*	aerial parts	[24]
120	Eremophiloside H	*A. eremophilus*	aerial parts	[24]
121	Eremophiloside I	*A. eremophilus*	aerial parts	[24]
122	Eremophiloside J	*A. eremophilus*	aerial parts	[24]
123	Eremophiloside K	*A. eremophilus*	aerial parts	[24]
124	Cyclomacroside A	*A. macropus*	roots	[58]
125	Bicusposide D	*A. bicuspis*	whole plant	[39]
126	3-*O*-[*α*-l-Arabinopyranosyl(1→2)-*β*-d-xylopyranosyl]-3*β*,6*α*,23*α*,25-tetrahydroxy-20(*R*),24(*R*)-16*β*,24;20,24-diepoxycycloartane	*A. campylosema* Boiss. *subsp. campylosema*	roots	[28]
127	Dihydrocycloorbigenin A	*A. orbiculatus*	aerial parts	[38]
128	Cycloorbigenin	*A. orbiculatus*	aerial parts	[38]
129	Cycloorbigenin B	*A. orbiculatus*	aerial parts	[38]
130	Cycloorbicoside A	*A. orbiculatus*	aerial parts	[38]
131	Cycloorbicoside B	*A. orbiculatus*	aerial parts	[38]
132	Cycloorbicoside C	*A. orbiculatus*	aerial parts	[38]
133	Cycloorbicoside G	*A. orbiculatus*	aerial parts	[38]
134	Tomentoside I	*A. tomentosus*	aerial parts	[59]
135	Deacetyltomentoside I	*A. tomentosus*	aerial parts	[59]
136	Tomentoside III	*A. tomentosus*	aerial parts	[59]
137	Tomentoside IV	*A. tomentosus*	aerial parts	[59]
138	Huangqiyenin E	*A. membranaceus*	leaves	[60]
139	Huangqiyenin F	*A. membranaceus*	leaves	[60]
140	Huangqiyegenin III	*A. membranaceus*	leaves	[60]
141	Huangqiyegenin IV	*A. membranaceus*	leaves	[60]
142	Trideacetylhuangqiyegenin III	*A. membranaceus*	leaves	[60]

Commonly, the aglycon of cycloartane-type saponins such as **1**–**62** possess an acyclic side chain at the 17-position. For the 17-acyclic side chain substituted cycloartane-type saponins, obtained from the Astragalus genus, the 24-position is often replaced by oxygen containing groups, and there are two kinds of steric configuration constituents with 24*S* (**1**–**28**) [6,9,10,11,12,13,14,15,16,17,18,19,20,21,22] or 24*R* (**29**–**48**) [7,14,15,16,17,23,24,25,26,27,28,29,30,31,32,33,34].

If there are hydroxyls substituted in the 20- and 24-positions, it is easy to dehydrate and form a furan ring between them. So the 20,24-epoxycycloartane-type saponins like **64**–**107** are derived from secondary metabolic pathways. Also there are usually 20*R*, 24*S* (**64**–**101**) [6,10,12,13,22,26,27,28,39,42,43,44,45,46,47,48,49,50,51,52,53] or 20*S*,24*R* (**102**–**107**) [54,55,56,57] steric configurations.

Some uncommon cycloartane triterpene glycosides such as **108**–**142 **were also isolated from the Astragalus genus. For example, the eremophilosides E–I (**118**–**121**) [24] are 16*β*,23-epoxycycloartanes. Among them, eremophilosides E (**118**) and F (**119**) are characterized as having an unusual loss of a four carbon side chain from C-24 to C-27 and a six-membered lactone ring between C-23 and C-16, while eremophilosides G–I (**117**, **120** and **121**) show an unusual modification of the cyclic side chain. On the other hand, eremophilosides J (**122**) and K (**123**) are 16*β*,23; 22*β*,25-diepoxycycloartanes, which are highly oxygenated cycloartane triterpenes.

In the study of isoprenoid plants of the Astragalus genus like *A. orbiculatus*, researchers found several derivatives of 16*β*,23,16*α*,24-diepoxycycloartane (**127**–**133**) from its aerial parts [38], which have been found only in this species.

Additionally, some cycloartane-type saponin ethylacetals (**134**–**142**) were identified from the extracts of Astragalus genus [59,60].

#### 2.1.2. Oleanane-Type Saponins

Apart from the cycloartane triterpene glycosides, many oleanane-type saponins shown in Table 2 were also isolated and identified from the Astragalus genus. Structure characterizations of this kind of saponin indicated they were substituted with a *β*-hydroxymethyl, instead of methyl in the 23-position.

**Table 2:** Oleanane triterpenoids from the Astragalus genus (**143**–**161**).

	Compound’s Name	Species Resource	Parts Used	Reference
143	3-*O*-[*α*-l-Rhamnopyranosyl(1→2)-*β*-d-xylopyranosyl(1→2)-*β*-d-glucuronopyranosyl]-21-*O*-*α*-l-rhamnopyranosyl-3*β*,21*β*,22*α*,24-tetrahydroxyolean-12-ene	*A. tauricolus*	whole plant	[61]
144	3-*O*-[*α*-l-Rhamnopyranosyl(1→2)-*β*-d-glucopyranosyl(1→2)-*β*-d-glucuronopyranosyl]-21-*O*-*α*-l-rhamnopyranosyl-3*β*,21*β*,22*α*,24-tetrahydroxyolean-12-ene	*A. tauricolus*	whole plant	[61]
145	3-*O*-[*α*-l-Rhamnopyranosyl(1→2)-*β*-d-glucopyranosyl(1→2)-*β*-d-glucuronopyranosyl]-3*β*,21*β*,22*α*,24,29-pentahydroxyolean-12-ene	*A. tauricolus*	whole plant	[61]
146	3-*O*-[*α*-l-Rhamnopyranosyl(1→2)-*β*-d-xylopyranosyl(1→2)-*β*-d-glucuronopyranosyl]-22-*O*-*α-*l*-*rhamnopyranosyl-3*β,*22*β,*24-trihydroxyolean-12-ene	*A. tauricolus*	whole plant	[61]
147	3-*O*-[*α*-l-Rhamnopyranosyl(1→2)-*β*-d-xylopyranosyl(1→2)-*β*-d-glucuronopyranosyl]-3*β*,21*β*,22*α*,24,29-pentahydroxyolean-12-ene	*A. angustifolius*	whole plant	[45]
148	3-*O*-[*α*-l-Rhamnopyranosyl(1→2)-*β*-d-xylopyranosyl(1→2)-*β*-d-glucuronopyranosyl]-3*β*,22*β*,24-trihydroxyolean-12-en-29-oic acid	*A. angustifolius*	whole plant	[45]
149	3-*O*-[*α*-l-Rhamnopyranosyl(1→2)-*β*-d-xylopyranosyl(1→2)-*β*-d-glucuronopyranosyl]-22-*O*-*α*-l-arabinopyranosyl-3*β*,22*β*,24-trihydroxyolean-12-ene	*A. angustifolius*	whole plant	[45]
150	29- *O*-*β*-d-Glucopyranosyl-3*β*,22*β,*24,29-tetrahydroxy-olean-12-ene	*A. angustifolius*	whole plant	[45]
151	Soyasapogenol B	*A. caprinus*	roots	[62]
		*A. bicuspis*	whole plant	[39]
152	3-*O*-[*β*-d-Xylopyranosyl(1→2)-*O*-*β*-d-glucopyranosyl(1→2)-*O*-*β*-d-glucuronopyranosyl] soyasapogenol B	*A*. *hareftae*	whole plant	[10]
153	3-*O*-*α*-l-Rhamnopyranosyl(1→2)-*β*-d-glucuronopyranosyl]-22-*O*-*β*-d-apiofuranosyl soyasapogenol B	*A. caprinus*	roots	[62]
154	3-*O*-[*α*-l-Rhamnopyranosyl(1→2)-*β-*d-xylopyranosyl(1→2)-*β*-d-glucuronopyranosyl]-29-*O*-*β*-d-glucopyranosyl-3*β*,22*β*,24-trihydroxyolean-12-en-29-oic acid	*A. tauricolus*	whole plant	[61]
155	3-*O*-[*α-*l-Rhamnopyranosyl(1→2)*-β-*d-glucopyranosyl(1→2)-*β*-d-glucuronopyranosyl]-29-*O*-*β*-d-glucopyranosyl-3*β*,22*β*,24,-trihydroxyolean-12-en-29-oic acid	*A. tauricolus*	whole plant	[61]
156	3-*O*-[*β*-d-Xylopyranosyl(1→2)-*β*-d-glucuronopyranosyl]-29-*O*-*β*-d-glucopyranosyl-3*β*,22*β*,24,-trihydroxyolean-12-en-29-oic acid	*A. tauricolus*	whole plant	[61]
157	3-*O*-[*α*-l-Rhamnopyranosyl-(1→2)-*β*-d-glucopyranosyl-(1→2)-*β*-d-glucuronopyranosyl]-29-*O*-*β*-d-glucopyranosyl-3*β*,22*β*,24,29-tetrahydroxyolean-12-ene	*A. tauricolus*	whole plant	[61]
158	3-*O*-[*α*-l-Rhamnopyranosyl-(1→2)-*β*-d-glucopyranosyl-(1→2)-*β-*d-glucuronopyranosyl]-3*β*,24-dihydroxyolean-12-ene-22-oxo-29-oic acid	*A. tauricolus*	whole plant	[61]
159	3-*O*-[*β*-d-Glucopyranosyl-(1→2)-*β*-d-glucuronopyranosyl]-29-*O*-*β*-d-glucopyranosyl-3*β*,22*β*,24,-trihydroxyolean-12-en-29-oic acid	*A. tauricolus*	whole plant	[61]
160	Azukisaponin V	*A. cruciatus*	aerial parts and roots	[2]
		*A*. *hareftae*	whole plant	[10]
161	Astragaloside VIII	*A. flavescens*	roots	[4]
		*A. cruciatus*	aerial parts and roots	[2]
		*A*. *hareftae*	whole plant	[10]
		*A. wiedemannianus*	whole plant	[13]
		*A. icmadophilus*	whole plant	[22]
		*A. angustifolius*	whole plant	[45]

### 2.2. Flavonoids

Just like many other herbs, Astragalus genus plants are also a rich source of flavonoids. The flavonoids in this genus include flavonols (**162**–**182**) [2,8,22,63,64,65,66,67,68], flavones (**183**–**193**) [2,8,53,63,66,69], flavonones (**194**–**195**) [65,70] and isoflavonoids (**196**–**221**) [16,50,53,65,66,70,71,72,73,74,75], which have many kinds of bioactivities.

In addition, some special flavonoids, such as sulfuretin (**222**) [65], isoliquiritigenin (**223**) [8], and pendulone (**224**) [50] have been obtained.

**Table 3:** Flavonoids from the Astragalus genus (**162**–**224**).

	Compound’s Name	Species Resource	Parts Used	Reference
162	Narcissin	*A. cruciatus*	aerial parts and roots	[2]
		*A. icmadophilus*	whole plant	[22]
		*A. corniculatus*	aerial parts	[63]
163	Nicotiflorin	*A. cruciatus*	aerial parts and roots	[2]
		*A. verrucosus*	aerial parts	[53]
		*A. asper*	aerial parts	[64]
164	Kaempferol 3-*O*-*α*-l-rhamnopyranosyl(1→4)-*α*-l-rhamnopyranosyl(1→6)-*β*-d-glucopyranoside	*A. cruciatus*	aerial parts and roots	[2]
165	Microcephalin I	*A. microcephalus*	leaves	[65]
166	Microcephalin II	*A. microcephalus*	leaves	[65]
167	Kaempferol-3-*O*-*α*-l-rhamnoxyloside	*A. microcephalus*	leaves	[66]
168	Quercetin	*A. asper*	aerial parts	[64]
		*A. corniculatus*	aerial parts	[63]
169	Quercimeritrin	*A. asper*	aerial parts	[64]
170	Rutin	*A. cruciatus*	aerial parts and roots	[2]
		*A. verrucosus*	aerial parts	[53]
		*A. asper*	aerial parts	[64]
171	Quercetin-3-*O*-*β*-d-glucopyranoside	*A. corniculatu*s	aerial parts	[63]
		*A. asper*	aerial parts	[64]
172	Kaempferol	*A. corniculatu*s	aerial parts	[63]
		*A. asper*	aerial parts	[64]
		*A. galegiformis*	leaves	[67]
173	Kaempferol-3-glucoside (Astragalin)	*A. asper*	aerial parts	[64]
		*A. galegiformis*	leaves	[67]
		*A. hamosus*	aerial parts	[68]
174	Isorhamnetin	*A. corniculatus*	aerial parts	[63]
		*A. hamosus*	aerial parts	[68]
175	Quercetin-3-*O*-galactoside	*A. corniculatus*	aerial parts	[63]
176	Quercetin-3,7-di-*β*-d-glucopyranoside-4'-*O*-*α*-l-rhamnopyranoside	*A. bombycinus*	whole plant	[8]
177	Quercetin-3,7-di-*O*-*β*-d-glucopyranoside	*A. bombycinus*	whole plant	[8]
178	Quercetin 3-*O*-*β*-d-glucopyranoside-7-*O*-*α*-l-rhamnopyranoside	*A. bombycinus*	whole plant	[8]
179	Flagaloside C	*A. galegiformis*	leaves	[67]
180	Flagaloside D	*A. galegiformis*	leaves	[67]
181	Kaempferol 3-*O*-robinobioside	*A. verrucosus*	aerial parts	[53]
182	7-*O*-Methyl-kaempferol-4'-*β*-d-galactopyranoside	*A. hamosus*	aerial parts	[68]
183	5,7,2'-Trihydroxyflavone	*A. cruciatus*	aerial parts and roots	[2]
184	Salvigenin	*A. propinquus*	roots	[69]
185	Apigenin	*A. bombycinus*	whole plant	[8]
		*A. verrucosus*	aerial parts	[53]
		*A. propinquus*	roots	[69]
186	Luteolin	*A. bombycinus*	whole plant	[8]
		*A. propinquus*	roots	[69]
187	7-Hydroxyflavone	*A. microcephalus*	leaves	[66]
188	5,2',4'-Trihydroxy-flavone-8-*C*-l-arabinopyranoside-7-*O*-*β*-d-glucopyranoside	*A. bombycinus*	whole plant	[8]
189	Apigenin 7-*O*-*β*-d-glucopyranoside	*A. bombycinus*	whole plant	[8]
190	Apigenin 7-*O*-gentobioside	*A. bombycinus*	whole plant	[8]
191	Luteolin 7-*O*-*β*-d-glucopyranoside	*A. bombycinus*	whole plant	[8]
192	Apigenin-8-*C*-glucoside (Vitexin)	*A. corniculatus*	aerial parts	[63]
193	Luteolin-8-*C*-glucoside (Orientin)	*A. corniculatus*	aerial parts	[63]
194	Eriodyctiol-7-*O*-glucoside	*A. corniculatus*	aerial parts	[63]
195	Liquiritigenin	*A. membranaceus*	roots	[70]
196	Odoration	*A. membranaceus* var. *mongholicus*	roots	[71]
197	Odoration-7-*O*-*β*-d-glucopyranoside	*A. mongholicus*	aerial parts	[72]
198	Calycosin-7-*O*-*β*-d-glucopyranoside	*A. ernestii*	roots	[16]
		*A. membranaceus*	roots	[70]
		*A. membranaceus* var. *mongholicus*	roots	[71]
		*A. mongholicus*	roots	[73]
		*A. membranaceus*	roots	[74]
199	Calycosin	*A. membranaceus*	roots	[70]
		*A. membranaceus* var. *mongholicus*	roots	[71]
		*A. mongholicus*	roots	[72]
		*A. membranaceus*	roots	[74]
200	Ononin	*A. membracaceus*	roots	[50]
		*A. verrucosus*	aerial parts	[53]
		*A. microcephalus*	leaves	[65]
		*A. mongholicus*	roots	[73]
		*A. membranaceus*	roots	[74]
201	Formononetin	*A. membranaceus*	roots	[70]
		*A. mongholicus*	roots	[72]
202	Calycosin 7-*O*-*β*-d-{6''-[(*E*)-but-2-enoyl]}-glucoside	*A. membracaceus*	roots	[50]
203	Pratensein 7-*O*-*β*-d-glucopyranoside	*A. membranaceus* var. *mongholicus*	roots	[71]
204	Pratensein	*A. verrucosus*	aerial parts	[53]
		*A. membranaceus* var. *mongholicus*	roots	[71]
205	Calycosin 7-*O*-*β*-d-(6''-acetyl)-glucoside	*A. membracaceus*	roots	[50]
206	6ꞌꞌ-Acetylononin	*A. membracaceus*	roots	[50]
207	Ammopiptanoside A	*A. membracaceus*	roots	[50]
208	7,5'-Dihydroxy-3'-methoxy-isoflavone-7-*O*-*β*-d-glucopyranoside	*A. membranaceus* var. *mongholicus*	roots	[71]
209	7-Hydroxy-3',5'-dimethoxyisoflavone	*A. peregrinus*	aerial parts	[75]
210	Daidzein	*A. bombycinus*	whole plant	[8]
		*A. verrucosus*	aerial parts	[53]
211	(3*R*,4*R*)-3-(2-Hydroxy-3,4-dimethoxy-phenyl)-chroman-4,7-diol-7-*O*-*β*-d-glucopyranoside	*A. membranaceus*	roots	[74]
212	(3*R*)-8,2'-Dihydroxy-7,4'-dimethoxyisoflavane	*A. membranaceus*	roots	[76]
213	(*R*)-3-(5-Hydroxy-2,3,4-trimethoxyphenyl)-chroman-7-ol	*A. membracaceus*	roots	[50]
214	Isomucronulatol 7-*O*-*β*-glucoside	*A. membracaceus*	roots	[50]
		*A. membranaceus*	roots	[70]
215	Isomucronulatol	*A. membracaceus*	roots	[50]
		*A. membranaceus*	roots	[70]
216	(–)-Methylinissolin 3-*O*-*β*-d-(6'-acetyl)-glucoside	*A. membracaceus*	roots	[50]
217	(–)-Methylinissolin 3-*O*-*β*-d-{6'-[(*E*)-but-2-enoyl]}-glucoside	*A. membracaceus*	roots	[50]
218	(–)-Methylinissolin 3-*O*-*β*-d-glucoside	*A. membracaceus*	roots	[50]
219	Licoagroside D	*A. membracaceus*	roots	[50]
220	Vesticarpan	*A. membracaceus*	roots	[50]
221	(–)-Methylinissolin	*A. membracaceus*	roots	[50]
222	Sulfuretin	*A. microcephalus*	leaves	[66]
223	Isoliquiritigenin	*A. membranaceus*	roots	[70]
224	Pendulone	*A. membracaceus*	roots	[50]

### 2.3. Polysaccharides

Yao *et al.*, analyzed the monosaccharide compositions for the Radix Astragali polysaccharide by gas chromatography, and identified the monosaccharides in it as arabinose, fructose, glucose, and mannose. Their molar ratios calculated according to the equation was 1:10.309:24.667:0.462 [77]. Xu *et al.*, isolated and purified two kinds of Astragalus polysaccharides (APS) (APS-I and APS-II) from the water extract of Radix Astragali. The research indicated that APS-I consisted of arabinose and glucose in the molar ratio of 1:3.45, with molecular weight 1,699,100 Da; meanwhile, APS-II consisted of rhamnose, arabinose and glucose in a molar ratio of 1:6.25:17.86 with molecular weight 1,197,600 Da [78].

### 2.4. Others

Undoubtedly, apart from the compounds mentioned above, others chemical constituents also exist in the Astragalus genus, including caffeic acid (**225**) [64], chlorogenic acid (**226**) [64], gentisin (**227**) [44], emodin (**228**) [44], 3-*O*-[*β*-d-apiofuranosyl(1→2)-*O*-*β*-d-glucopyranosyl] maltol (**229**) [27], *β*-sitosterol (**230**) [16,30,51,73] and *β*-sitosterol *β*-d-glycopyranoside (**231**) [51].

Certainly, some other constituents, such as amino acids and proteins have been obtained from Astragalus genus plants, which were also found to be rich in metal elements like Ca, Mg, Fe, Cu, Zn, and Mn [79].

## 3. Biological Activities of the Astragalus Genus

*A. membranaceus*, *A. mongholicus* and *A. complanatus* have been mainly used in folk medicine for their anti-inflammatory, immunostimulant, antioxidative, anti-cancer, antidiabetic, cardioprotective, hepatoprotective, and antiviral properties in recent years. The active constituents for the above-mentioned effects were proved to be polysaccharides, saponins, and flavonoids.

### 3.1. Anti-Inflammatory Activity

Astragalus extract, along with its polysaccharides, and saponins showed anti-inflammatory activity both *in vitro* and *in vivo*. Kim *et al.*, found that the extract of *A. membranaceus* not only improved the atopic dermatitis skin lesions in 1-chloro-2,4-dinitrobenzene-induced mice as well as restoring nuclear factor-κB expression markedly, but also suppressed the expression of Th2 cytokines and significantly decreased the TNF-*α* level. They then figured out that *A. membranaceus* was effective for treating atopic dermatitis by regulating cytokines [80]. Ryu *et al.*, verified that Astragali Radix had an anti-inflammatory effect mediated by the MKP-1-dependent inactivation of p38 and Erk1/2 and the inhibition of NFkappaB-mediated transcription [81]. As the main composition of Astragalus, Astragalus polysaccharides can effectively ameliorate the palmitate-induced pro-inflammatory responses in RAW264.7 cells through AMPK activity [82]. They also showed anti-inflammatory activity, along with structure protective properties for lipopolysaccharide-infected Caco2 cells [83]. On the other hand, the anti-inflammatory activity of saponins was also studied. The results, of agroastragalosides I, II, isoastragaloside II, and astragaloside IV showed the ability to inhibit lipopolysaccharide-induced nitric oxide production in RAW264.7 macrophages [84]. Meanwhile, astragaloside IV was shown to be a promising natural product with both healing and anti-scar effects for wound treatment [85], could be used as a novel anti-inflammatory agent, and attenuated diabetic nephropathy in rats by inhibiting NF-κB mediated inflammatory gene expression [86].

### 3.2. Immunoregulatory Activity

Qin *et al.*, studied the effect of *A. membranaceus* extract on the advanced glycation end product-induced inflammatory response in *α*-1 macrophages. The results suggested that it could inhibit advanced glycation end product-induced inflammatory cytokine production to down-regulate macrophage-mediated inflammation via p38 mitogen-activated protein kinase and nuclear factor (NF)-κB signaling pathways [87]. Du *et al.*, investigated the potential adjuvant effect of Astragalus polysaccharides on humoral and cellular immune responses to hepatitis B subunit vaccine. The result suggested that it was a potent adjuvant for the hepatitis B subunit vaccine and could enhance both humoral and cellular immune responses via activation of the Toll-like receptor 4 signaling pathway and inhibit the expression of transforming growth factor *β* [88]. Nalbantsoy *et al.*, studied the *in vivo* effects of two Astragalus saponins on the immune response cytokines by using six to eight week old male Swiss albino mice. The results showed that astragaloside VII and macrophyllosaponin B showed powerful immunoregulatory effects without stimulation of inflammatory cytokines in mice, and had no significant effect on the inflammatory cellular targets *in vitro* [89]. Huang *et al.* found that astragaloside IV could rival the suppressing effect of high mobility group box 1 protein on immune function of regulatory T cells with dose-dependent *in vitro* [90].

### 3.3. Anti-Tumor Activity

Recently, many active screening results have indicated that Astragalus polysaccharides, saponins, and flavonoids have anti-tumor activities. Tian *et al.*, investigated the adjunct anticancer effect of Astragalus polysaccharides on H22 tumor-bearing mice, and found that it exerted a synergistic anti-tumor effect with adriamycin and to alleviate the decrease in the sizes of the spleen and thymus induced by adriamycin in H22 tumor-bearing mice [91]. As a potential anti-tumor saponin, astragaloside IV could down-regulate Vav3.1 expression in a dose- and time-dependent manner [92]. Meanwhile, astragaloside II could down regulate the expression of the P-glycoprotein and mdr1 gene, which suggested it was a potent multidrug resistance reversal agent and could be a potential adjunctive agent for hepatic cancer chemotherapy [93]. On the other hand, the experimental data showed that the total flavonoids of Astragalus and calycosin could inhibit the proliferation of K562 cells [94].

### 3.4. Cardioprotection Activity

Ma *et al.*, studied the cardio protective effect of the extract of Radix Astragali on myocardial ischemia and its underlying mechanisms in ROS-mediated signaling cascade *in vivo* by using different rat models, and drew the conclusion that the cardio protection was due to a protection of tissue structure and a decrease in serum markers of the ischemic injury [95]. The total flavonoids of *A. mongholicus* are the active components, which benefit cardiovascular disease attributed to the potent antioxidant activity in improving the atherosclerosis profile [96]. Isoflavones, calycosin and formononetin from the Astragalus root, could promote dimethylarginine dimethylaminohydrolase-2 protein and mRNA expressions in Madin Darby Canine Kidney (MDCK) II cells, and up regulate the neuronal nitric oxide synthase levels [97]. Astragaloside IV could prolong the action potential duration of guinea-pig ventricular myocytes, which might be explained by its inhibition of K^+^ currents [98].

### 3.5. Antidiabetic

The study of Liu *et al.*, indicated that Astragalus polysaccharide could regulate part of the insulin signaling in insulin-resistant skeletal muscle, and could be a potential insulin sensitizer for the treatment of type 2 diabetes [99]. Zhou *et al.*, found Astragalus polysaccharide could up regulate the expression of galectin-1 in muscle of type I diabetes mellitus mice [100]. Saponins and astragaloside IV could exert protective effects against the progression of peripheral neuropathy in streptozotocin-induced diabetes in rats [101]. In addition, astragaloside V was found to inhibit the formation of *N*-(carboxymethyl)lysine and pentosidine during the incubation of bovine serum albumin with ribose, which suggested that it might be a potentially useful strategy for the prevention of clinical diabetic complication by inhibiting advanced glycation end products [102].

### 3.6. Anti-Oxidative Activity

The anti-oxidative activities of some flavonoids and saponins from *A. mongholicus* have been studied. For example, formononetin, calycosin, calycosin-7-*O*-*β*-d-glucoside could scavenge 1,1-diphenyl-2-picrylhydrazyl free radicals *in vitro*. Formononetin and calycosin were found to inhibit xanthine/xanthine oxidase-induced cell injury significantly. Among them, calycosin exhibited the most potent antioxidant activity both in the cell-free system and in the cell system [73]. The compound 7,2-dihydroxy-3',4'-dimethoxyisoflavan-7-*O*-*β*-d-glucoside and calycosin-7-*O*-*β*-d-glucoside from *A. membranaceus* showed anti-lipid peroxidative activities [103]. The saponin, astragaloside IV can inhibit hepatic stellate cells activation by inhibiting generation of oxidative stress and associated p38 MAPK activation [104].

### 3.7. Anti-Aging

According to the study of the anti-aging effect of astragalosides, Lei *et al.*, suggested that the mechanism might be related to the improvement of brain function and immunomodulatory effects [105]. Gao *et al.*, concluded that Astragalus polysaccharides could lengthen the living time of mice, improve the activity of superoxide dismutase and decrease the malonaldehyde content in mice blood serum compared with the control group, which suggested that Astragalus polysaccharides have anti-aging effects [106].

### 3.8. Other Biological Activities

Additionally, according to previous research, the plants of Astragalus species also have pharmacological effects such as antiviral, hepatoprotective, neuron protective, and so on.

## 4. Analyses

Researchers have conducted numbers of qualitatively and quantitatively analytical experiments on the plants in the Astragalus genus by different methods. Among them, the analyses of flavonoids and saponins from the radix of *A. membranaceus* and *A. mongholicus* were well done.

Han *et al.*, studied ultra-performance liquid chromatography for quantification of flavone in *A. membranaceus*, the outcome of which showed that the linear ranges of calycosin glycoside, formononetin glycoside, calycosin, and formononetin were 0.1313–1.313 g/L (r = 0.9997), 0.1186–1.186 g/L (r = 0.9994), 0.0206–0.206 g/L (r = 0.9995), and 0.0150–0.150 g/L (r = 0.9995), respectively. The average recoveries were 97.07%, 97.26%, 97.45% and 96.97% respectively [107]. The research results of Zhang *et al.*, indicated that the content of flavonoids in Radix Astragali of different growth years increased along with the growth period, and the types of flavonoids remained the same [108]. By comparing the retention time and MS data with those obtained from the authentic compounds and the published data, Ye *et al.*, identified a total of 19 compounds including 11 isoflavonoids and eight saponins [109].

In addition, analyses for other constituents in *A. membranaceus* and *A. mongholicus* and composition analysis for other species of Astragalus were also conducted. Huang *et al.*, studied the water extract of Radix Astragali by infrared spectroscopy, and found it contained an abundance of polyose, and the residue from it included some substances such as starch, cellulose, and lignin. The study provided an effective reference for the further analyses of chemical components and the improvement of extraction-separation technologies of TCM [110]. Using solid phase microextraction followed by GC-MS analysis, Movafeghi *et al.*, identified the volatile organic compounds in the leaves, roots and gum of *A. compactus*. As a result, a range of volatile compounds were recognized in different parts of it under optimized conditions, but tetradecane 1-chloro only existed in roots [111]. Sun *et al.*, determined the chemical components of *A. hamiensis* with a systematic extract method and TLC, the results showed that it contained alkaloids, polysaccharides, glucosides, amino acids, steroids, terpenoids, oils, tannins, phenols, organic acids, flavonoids-chinones, cardiac glycosides, and coumarins. Futhermore, they found the alkaloids were mainly swainsonine and analogous indolizidine. Meanwhile, *A. hamiensis* was found to contain small amount of ermopsine and anagyrine, which belong to quinolizidine [112].

## 5. Conclusions

As a TCM, the root of the Astragalus plant, Huang Qi, has been used in Chinese medicine for thousands of years. There are over two thousand species of Astragalus, among which only *A. membranaceus* and *A. mongholicus* are primarily used for medicinal purposes. In light of the considerable interest generated in the chemistry and pharmacological properties of the Astragalus plant, this review summarizes the retrieved literature published over the last 10 years dealing with several aspects including phytochemistry, bioactivity, and the research of the analysis.

In the field of phytochemistry and analysis, the chemical constituents from 46 kinds of Astragalus species have been studied. Although more than 200 compounds, including cycloartane-type saponins and flavonoids, were obtained from them, the systematic phytochemistry research needs to be improved. In addition, though the researchers conducted a number of qualitatively and quantitatively analytical experiments for flavonoids and saponins from the radix of *A. membranaceus* and *A. mongholicus* by different methods, the simultaneous analysis of two kinds of constituents was rarely found. In the field of pharmacology, the bioactivity evaluation of extracts and isolated compounds focused on anti-inflammatory, immunostimulant, antioxidative, anti-cancer, antidiabetic, cardioprotective, hepatoprotective, and antiviral, but anti-inflammation activity research of flavonoids was rare. Though a lot of results of pharmacological studies were carried out using crude extract of Astragalus species, the relationship between chemical constituents and activity is still unclear. Additionally, data on pharmacokinetics and bioavailability, especially related to the target tissue, need to be further supplemented.
